# Dose-Response and Substitution Analyzes of Sweet Beverage Consumption and Body Weight in Dutch Adults: The Lifelines Cohort Study

**DOI:** 10.3389/fnut.2022.889042

**Published:** 2022-06-24

**Authors:** Marion E. C. Buso, Elske M. Brouwer-Brolsma, Novita D. Naomi, Joanne A. Harrold, Jason C. G. Halford, Anne Raben, Edith J. M. Feskens

**Affiliations:** ^1^Division of Nutrition and Health, Wageningen University and Research, Wageningen, Netherlands; ^2^Department of Psychology, University of Liverpool, Liverpool, United Kingdom; ^3^School of Psychology, University of Leeds, Leeds, United Kingdom; ^4^Department of Nutrition, Exercise and Sports, Faculty of Science, University of Copenhagen, Denmark and Clinical Research, Copenhagen University Hospital - Steno Diabetes Center Copenhagen, Herlev, Denmark

**Keywords:** waist circumference, overweight, abdominal obesity, population study, non-calorie sweeteners

## Abstract

**Background/Methods:**

Prospective studies investigating sweet beverages and body weight associations show inconsistent results. Within the SWEET project, we examined prospective dose-response associations of sugar-sweetened beverages (SSB), low/no-calorie beverages (LNCB), and fruit juice with body weight-related outcomes among 78,286 Dutch adults followed for ~4 years. Baseline intakes were assessed using a validated food-frequency questionnaire (FFQ) with 150 ml representing a standard serving. Outcome variables were body weight change, waist circumference change, overweight/obesity, and abdominal obesity. Associations were investigated by using linear and non-linear dose-response analysis, as well as substitution models while adjusting for multiple socio-demographic, lifestyle, health, and dietary variables.

**Results:**

Participants were 46 ± 13 (mean ± SD) years old and 60% were women. Adjusted dose-response analyzes indicated an association between SSB and LNCB, and both body weight (+0.02 kg/year; SE 0.01 and +0.06 kg/year; SE 0.01) and waist circumference changes (+0.04 cm/year; SE: 0.01 and +0.11 cm/year; SE: 0.01). Associations for overweight/obesity and abdominal obesity incidence were +3% (95%CI: 1.00–1.06) and +2% (95%CI: 0.99–1.06) for SSB and +8% (95%CI: 1.06–1.11) and +5% (95%CI: 1.03–1.07) for LNCB, respectively. Substitution of SSB with LNCB was associated with higher weight change (+0.04 kg/year), waist circumference change (+0.09 cm/year), overweight/obesity incidence (+6%), but not abdominal obesity incidence. For fruit juice, we observed beneficial associations for intake levels below ~1 serving/day with weight, waist circumference change, and overweight/obesity incidence, and no association with abdominal obesity. Subsequent substitution analyzes indicated a small beneficial association for the replacement of SSB with fruit juice on weight (−0.04 kg/year) and waist circumference (−0.04 cm/year), but not with other outcomes.

**Conclusions:**

Overall, our results suggest that habitual consumption of both SSB and LNCB may adversely affect weight-related outcomes. In contrast, fruit juice consumption <150 ml may be beneficial with respect to weight and waist circumference.

## Introduction

From 1975 to 2016, the prevalence of obesity among adults increased from 100 million to 671 million worldwide (1). As obesity has been associated with major health consequences, such as type 2 diabetes and cardiovascular diseases (2), effective interventions to decrease prevalence of overweight and obesity are urgently needed. Weight gain has been partly attributed to higher caloric intakes, including the consumption of added sugar in the form of high-calorie sugar-sweetened beverages (SSB) (3–5). Consequently, major efforts have been made to replace sugars with low/no-calorie sweeteners such as aspartame, acesulfame-K, saccharin, and sucralose (6), and to develop low/no-calorie beverages (LNCB) (7, 8).

Despite the large body of research on alternatives for SSB, their impact on body weight remains a topic of sustained debate due to inconsistent findings. Available randomized control trials (RCTs) and meta-analyzes of RCTs on weight loss generally indicate a beneficial impact of consuming LNCB instead of SSB (9–13). In contrast, meta-analyzes of observational studies observed either no association (9) or a modest positive association between LNCB consumption and body mass index (BMI) (12, 13). Similar inconsistencies have also been observed for fruit juice (14, 15). Thus, although limited SSB consumption is recommended, there is insufficient evidence on whether or not LNCB and fruit juice could serve as healthier alternatives.

The conflicting findings in current literature may be explained by several methodological aspects. Although RCTs have better internal validity and are often considered superior over observational studies, the majority of the RCTs had small sample sizes and were short-term (≤6 months) (9, 13). Here, observational studies offer the benefit to explore long-term associations between SSB, LNCB, fruit juice, and body weight. Nevertheless, so far only few observational studies explored both linear and non-linear dose-response associations of SSB and LNCB consumption with weight-related outcomes (16, 17). Exploration of non-linear dose-response associations for fruit juice and weight-related outcomes is even lacking while such associations have been reported for fruit juices, cardiovascular diseases (CVD), and type 2 diabetes (18, 19). Moreover, existing large-scale prospective studies on body weight mostly used self-reported measures, and few investigated outcomes beyond body weight such as waist circumference. Finally, only a limited number of studies have conducted substitution analyzes to investigate the replacement of SSB with other beverages on body weight (20–22).

Therefore, we examined prospective dose-response associations of habitual SSB, LNCB, and fruit juice consumption with measured changes in body weight and waist circumference, and incidence of overweight/obesity and abdominal obesity, and evaluated the theoretical substitution of SSB with LNCB and fruit juices while using data of 78,286 Dutch adults followed for ~4 years.

## Materials and Methods

### The SWEET Project

The SWEET project is a Horizon 2020 funded project that aims to develop and review evidence on long-term benefits and potential risks involved with replacing sugars with low or non-calorie sweeteners and sweetness enhancers in the context of public health and safety, obesity, and sustainability (https://sweetproject.eu/). The current study using data from the Lifelines Cohort Study was conducted as part of a work package that aims to investigate long-term associations between sweeteners and health outcomes in population studies.

### Study Population and Design

The Lifelines Cohort Study is a multi-disciplinary prospective population-based cohort study examining in a unique three-generation design, the health and health-related behaviors of 167,729 persons living in the North of The Netherlands, including children (0–18 years old), adults (18–65 years old) and older adults (>65 years old) (23). It employs a broad range of investigative procedures in assessing the biomedical, socio-demographic, behavioral, physical, and psychological factors which contribute to the health and disease of the general population, with a special focus on multi-morbidity and complex genetics. Participants were recruited between 2006 and 2013 and will be followed for over 30 years. Potential participants with severe psychiatric or physical illness, limited life expectancy (<5 years), or insufficient knowledge of the Dutch language were not eligible for participation. Every 1.5 years, participants are invited to complete a follow-up questionnaire, and on average every 5 years, several physical measurements are performed and additional questionnaires are administrated. At the time of the current analysis, baseline data of 152,728 adults were available. After excluding those with unreliable dietary data, i.e., total energy intakes <500 and >3,500 kcal/day for women and <800 and >4,000 kcal/day for men (24, 25), 128,612 adults were included of which 84,545 had data on body weight and waist circumference change. A total of 78,286 adults met the inclusion criteria for the prospective analysis after the exclusion of missing data for covariates (Supplementary Figure 1). These 78,286 participants had their first follow-up exam between 1 and 9 years after baseline, with a median of 4 years after baseline. The Lifelines Cohort Study was conducted in accordance with the principles of the Declaration of Helsinki and the research code University Medical Center Groningen (UMCG). The Lifelines Cohort Study has been approved by The Medical Ethical Review Committee of the University Medical Center in Groningen. All participants provided written informed consent before participation.

### Anthropometry

Measurements of body weight, waist circumference, and BMI were carried out at baseline and follow-up by trained professionals. Body weight was measured to the nearest 0.1 kg with a digital scale (SECA 761) after participants were asked to wear light clothing and remove shoes. Height and waist circumference were measured to the nearest 0.1 cm, with a stadiometer (SECA 222) and measuring tape (SECA 200), respectively. BMI was obtained by dividing the weight of participants by height squared (kg/m^2^). Weight change (kg/year) and waist circumference change (cm/year) were calculated by subtracting the baseline measure to the follow-up measurements and dividing by the follow-up time, (i.e., weight follow-up – weight baseline)/years of follow-up). Incidence of overweight/obesity were defined by a BMI ≥25 kg/m^2^ at follow-up and abdominal obesity with a waist circumference >94 cm for men and >80 cm for women, based on the World Health Organization (WHO) cuts offs points (26). Additionally, participants were asked whether they wanted to lose weight, which we interpreted as “desire to lose weight (yes/no).”

### Dietary Assessment

In the Lifelines Cohort Study, dietary intake was assessed with a semi-quantitative 110-item food-frequency questionnaire (FFQ). A detailed description of the FFQ can be found elsewhere (23, 27). In short, average energy and nutrient intakes were calculated by multiplying the frequency of consumption by portion size and nutrient content per gram using the 2011 Dutch food composition table (28). For the current analyzes, SSB was defined as soda sugar drinks or lemonade (both carbonated and non-carbonated). Fruit juice corresponded to 100% fruit juice and other fruit drinks. LNCB was defined as all items covering “diet soda or light soda.” Baseline measurement of the diet was performed between 2006 and 2013. For this analysis, a standardized serving of 150 g (~150 ml) was calculated in all studies based on the smallest standard packaging for soft drinks.

### Covariates

Covariates, including age (years), sex (men/women), educational level (low, medium, or high), smoking status (never, former or current), and medical history (yes/no), were assessed with either self- or interview-administered questionnaires (23). Educational level was categorized into less than secondary school qualification (low), secondary school diploma up to university classes but no Bachelor's degree (medium), and Bachelor, Master or PhD degree (high). Participant history of diseases (type 2 diabetes, CVD, hypertension, and hypercholesterolemia) were assessed by self-report or medical staff at recruitment and subsequent visits. Physical activity was assessed using the Short Questionnaire to Assess Health (SQUASH) (29) and the Activity Questionnaire for Adults and Adolescents (AQuAA) (24) and physical activity is thus reported as MET-min/week for light, moderate and intense exercise and in min/week for sedentary behavior (i.e. watching TV).

### Statistical Analyzes

Baseline characteristics are presented by mean (SD), median (25th, 75th percentile), or *n* (%) where appropriate. To evaluate the nature of the dose-response relationships between beverages and weight related-outcomes, restricted cubic spline analysis (three knots) was performed (30). The fit of the spline model was tested against a linear model with a likelihood-ratio test. To evaluate the association between beverage consumption and weight and waist circumference changes, multiple linear regression was used. To evaluate the associations between beverage consumption and incidence of overweight/obesity and abdominal obesity, Cox proportional hazards regression with robust variance estimation and a constant follow-up time was used to obtain unbiased incidence proportion ratios (IPR) (31, 32). To investigate the association with weight-related outcomes when replacing each serving of SSB with a serving of either LNCB or fruit juice, theoretical substitution analyzes were conducted by means of a leave-one-out model (33). This model included the sum of all beverages as one variable followed by the beverages defined as replacement, as well as all other covariates as modeled in the analyzes. In a sub-sample of Lifelines where water consumption was available (*N* = 22,859), we additionally studied the replacement of SSB and LNCB with water for comparaison purposes. In all models, potential confounders were identified based on a priori knowledge. Models were adjusted for sex and age (model 1) + height and baseline weight (or baseline BMI for overweight/obesity incidence models) or baseline waist circumference (for models with waist circumference or abdominal obesity as outcome; model 2), + education (low, medium, and high), physical activity (light, moderate and intense in METs-min/week), sedentary behavior (min/week), alcohol intake (ethanol categories: non-consumers, ≤10 g, >10–20 g, and >20 g/day), smoking (never, former or current), dietary variables (g/day), namely meat, dairy, legumes, vegetables, nuts, fruits, potatoes, fats, grains, tea, coffee, sugary food intakes and other beverages (servings/day, i.e., SSB adjusted for fruit juice and LNCB and vice-versa) and history of diseases (self-reported diabetes and history of CVD, hypertension, and hypercholesterolemia; model 3). As total energy may mediate and thus attenuate the associations under investigation, particularly in the case of SSB and fruit juice, the final models were tested with and without adjustment for total energy intake (model 4). Additional analyzes were performed adjusting for desire to lose weight (yes/no) and sensitivity analyzes were conducted by excluding participants with any self-reported health conditions at baseline (i.e., diabetes type 2, CVD, hypercholesterolemia, or hypertension). We also tested the interaction for BMI (<25 and ≥25 kg/m^2^), sex and age (<46 years old and ≥46 years old) and studied the stratified data accordingly. All analyzes were performed using R 3.6.1 and RStudio 1.0.

## Results

Participants (*n* = 78,286) had a mean age of 45.9 (SD 12.7) years and 60% were women (Table 1). Baseline mean BMI was 26.0 (SD 4.2) kg/m^2^ and 45% of the participants had a normal BMI <25 kg/m^2^. Mean body weight change during follow-up was +0.02 (SD 1.58) kg/year and mean waist circumference change was +0.01 (SD 2.04) cm/year. On average, participants with normal BMI gained weight and waist circumference [+0.21 (SD 1.20) kg/year and + 0.10 (SD 1.88) cm/year], while participants with higher BMI lost weight and waist circumference [−0.13 (SD 1.82) kg/year; and −0.07 (SD 2.15) cm/year]. Participants with overweight/obesity also reported a higher desire to lose weight at baseline (79 vs. 29% in participants with normal BMI). Of the 35,202 participants with normal BMI at baseline, 4,884 (14%) developed overweight or obesity and out of the 31,292 participants with normal waist circumference at baseline, 6,896 (22%) developed abdominal obesity (Table 1).

**Table 1:** General characteristics of the Lifelines Cohort Study.

**Characteristics** ^**a**^	**Overall**	**BMI <25 kg/m^2^**	**BMI ≥25 kg/m^2^** ^**b**^
*N*	78,286	35,202	43,084
Women, *n* (%)	46,663 (59.6)	23,617 (67.1)	22,046 (53.5)
Age, years	45.9 (12.7)	43.1 (12.9)	48.1 (12.1)
**Education**, ***n*** **(%)**			
Low	3,077 (3.9)	1,073 (3.0)	2,004 (4.7)
Intermediate	50,690 (64.7)	20,931 (59.5)	29,759 (69.1)
High	24,519 (31.3)	13,198 (37.5)	11,321 (26.3)
Height (cm)	174.7 (9.3)	174.6 (9.0)	174.8 (9.5)
Body weight, kg	79.5 (15.0)	69.1 (9.1)	88.1 (13.3)
Waist circumference, cm	90.1 (12.2)	81.3 (7.7)	97.3 (10.3)
BMI, kg/m^2^	26.0 (4.2)	22.6 (1.7)	28.8 (3.5)
Desire to lose weight^c^	44,411 (56.8)	10,331 (29.4)	34,080 (79.2)
**Physical activity (METs min/week)**			
Intense	0 [0,630]	0 [0, 840]	0.0 [0, 420]
Moderate	1,665 [806, 2,948]	1,605 [788, 2,847]	1,702 [818, 3,045]
Sedentary (min/week)	840 [630,1,260]	840 [630, 1,260]	1,050 [840, 1,470]
**Smoking**, ***n*** **(%)**			
Never	36,461 (46.6)	18,020 (51.2)	18,441 (43.8)
Former	27,376 (35.0)	10,337 (29.4)	17,039 (39.5)
Current	14,449 (18.5)	6,845 (19.4)	7,604 (17.6)
**Alcohol (ethanol) intake**, ***n*** **(%)**			
No alcohol	1,919 (2.5)	702 (2.0)	1,217 (2.8)
Medium (0– ≤ 10g)	55,888 (71.4)	25,925 (73.6)	29,963 (69.5)
High (10– ≤ 20g)	15,032 (19.2)	6,593 (18.7)	8,439 (19.6)
Very high (>20 g)	5,447 (7.0)	1,982 (5.6)	3,465 (8.0)
Total energy, g/day	1,977 [1,640, 2,380]	1,997 [1,665, 2,387]	1,959 [1,619, 2,373]
SSB servings/day	0.11 [0.0, 0.62]	0.14 [0.00, 0.63]	0.09 [0.00, 0.60]
LNCB servings/day	0.07 [0.0, 0.61]	0.04 [0.00, 0.36]	0.12 [0.0, 0.71]
Fruit Juice servings/day	0.18 [0.04, 0.64]	0.18 [0.04, 0.71]	0.18 [0.00, 0.64]
Type 2 diabetes, *n* (%)	1,853 (2.4)	297 (0.8)	1,556 (3.6)
CVD, *n* (%)	1,805 (2.3)	541 (1.5)	1,264 (2.9)
Hypertension, *n* (%)	17,499 (22.4)	4,841 (13.8)	12,647 (29.4)
Hypercholesterolemia, *n* (%)	11,070 (14.1)	3,226 (9.2)	7,834 (18.2)
Body weight change (kg/year)	0.02 (1.58)	0.21 (1.20)	−0.13 (1.82)
Waist circumference change (cm/year)	0.01 (2.04)	0.10 (1.88)	−0.07 (2.15)
Overweight/obesity incidence	–	4,884/35,202 (13.9)	–
Abdominal obesity incidence	–	6,896/31,292 (22.0)	–

a *Mean (SD), median [25th-75th percentile] or n (%)*.

b *All P-values for the difference between BMI categories were < 0.01*.

c *Data was missing for 134 participants*.

*BMI, body mass index; SSB, sugar-sweetened beverages; LNCB, low/no-calorie beverages; CVD, cardiovascular diseases*.

Overall, 62% of participants consumed SSB, 57% LNCB, and 77% fruit juice. Median [25th−75th percentile] baseline intakes were 0.1 [0.0; 0.6] servings/day for SSB and LNCB, and 0.2 [0.0; 0.6] servings/day for fruit juices. In general, those consuming the highest levels of SSB and fruit juice were more likely to be men, were younger, had less often a chronic disease history, and had a slightly lower BMI and higher energy intake (all *P*-trend < 0.001; Supplementary Table 1). Concerning LNCB, those consuming the highest levels were more likely to be women, to be younger, and to have a higher BMI (all *P*-trend < 0.001). SSB and fruit juice consumption were both positively correlated with energy intake (*r* = 0.34 and *r* = 0.21 respectively; *P* < 0.001), while no correlation was observed between LNCB use and energy intake (*r* = 0.005).

**Figure 1:**
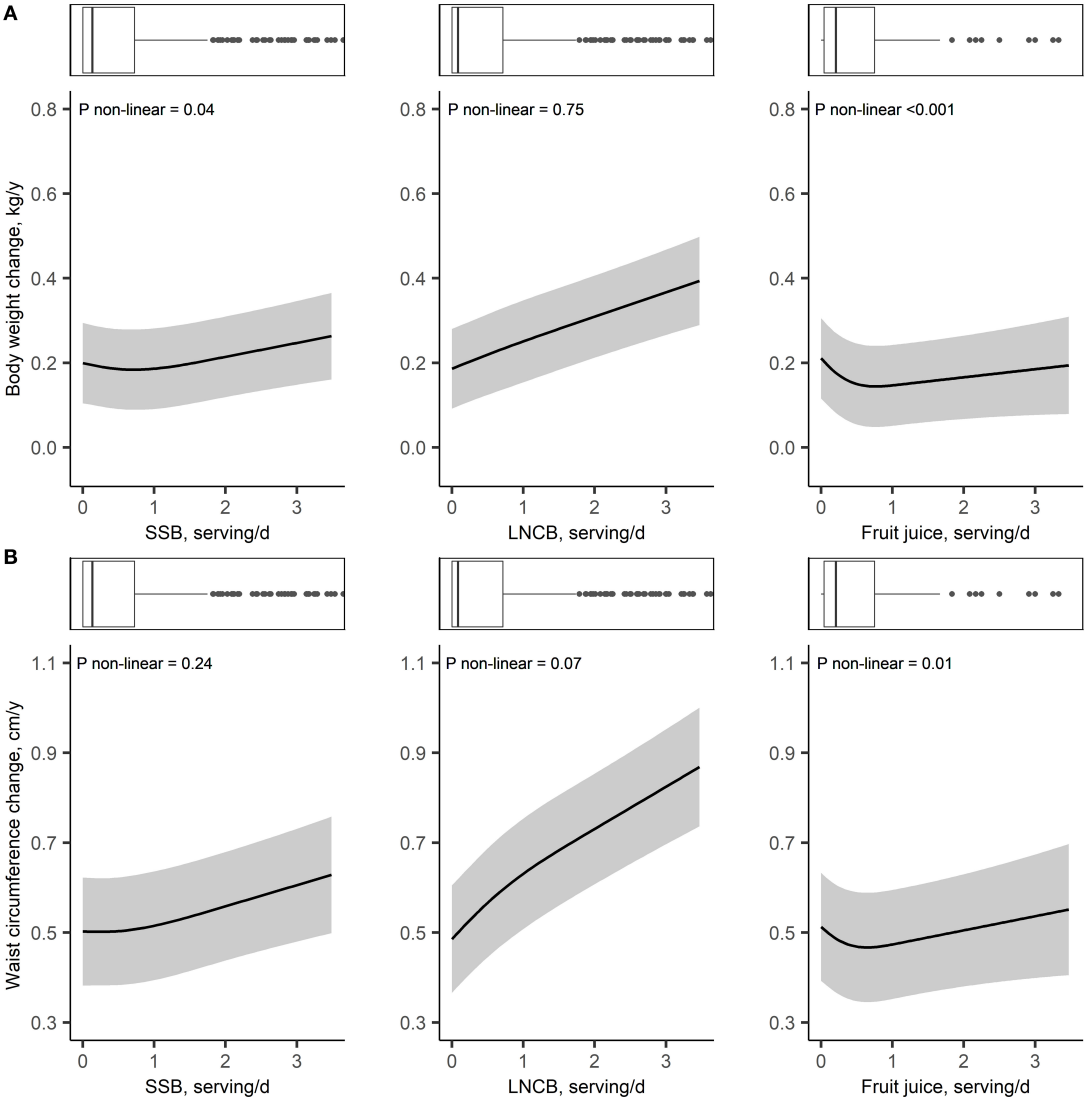
Adjusted dose-response associations of SSB, LNCB, and fruit juice consumption with body weight change (kg/year) **(A)** and waist circumference change (cm/year) **(B)** in the Lifelines Cohorts Study; Models were adjusted for age, sex, height, baseline weight or baseline waist circumference (for models with waist circumference as outcome), education, alcohol intake, smoking, physical activity, all dietary factors, total energy intake and history of diseases. SSB, sugar-sweetened beverages; LNCB, low/no-calorie beverages.

**Figure 2:**
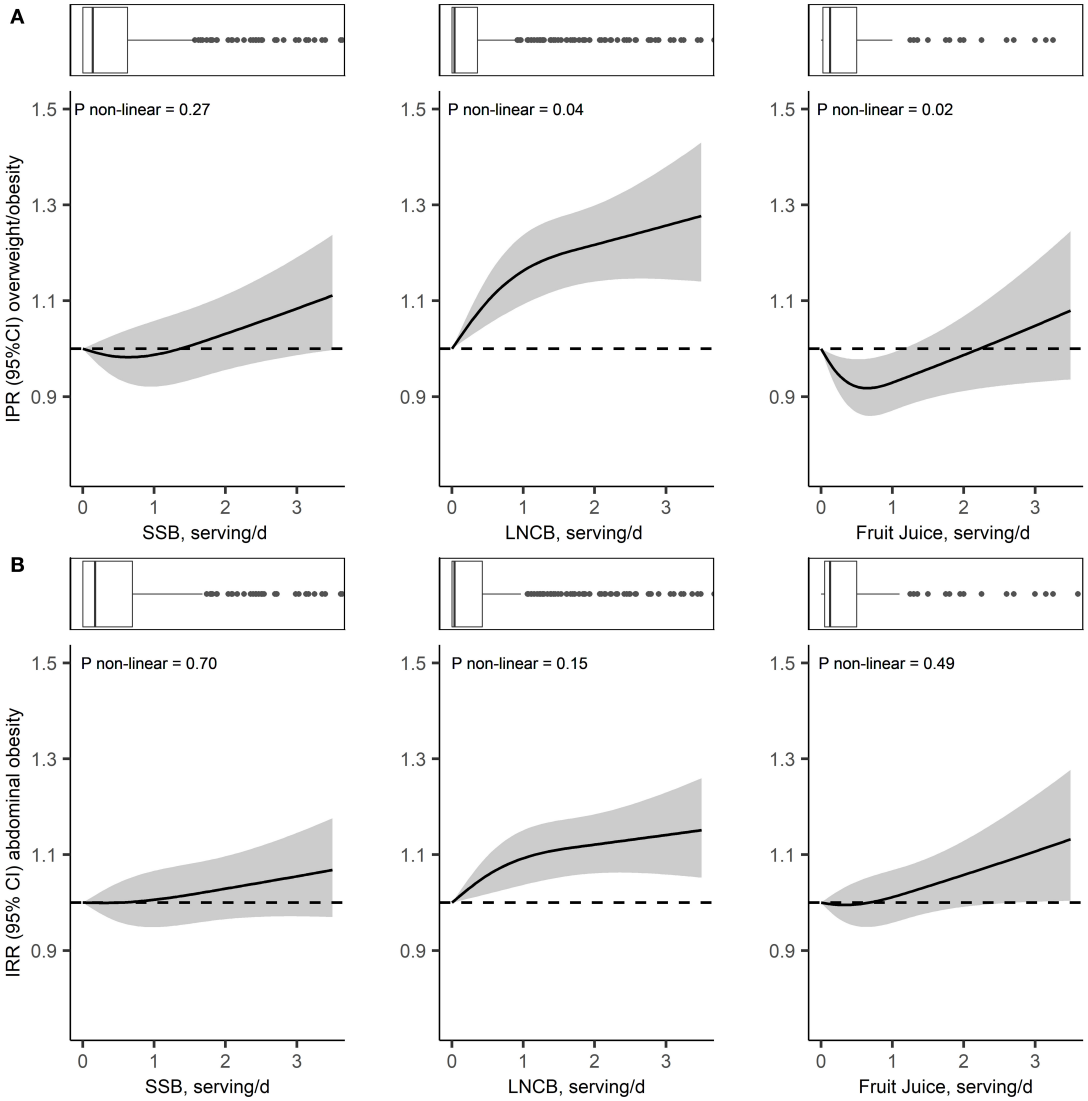
Adjusted dose-response associations of SSB, LNCB, and fruit juice consumption with overweight/obesity incidence **(A)** and abdominal obesity incidence **(B)** in participants with normal values at baseline (i.e., <25 kg/m^2^ for overweight/obesity and ≤94 cm in men and ≤80cm in women for abdominal obesity) in the Lifelines Cohort Study; Models were adjusted for age, sex, baseline BMI or baseline waist circumference and height (for models with abdominal obesity as outcome), education, alcohol intake, smoking, physical activity, all dietary factors, total energy intake and history of diseases; SSB, sugar-sweetened beverages; LNCB, low/no-calorie beverage; IPR, incidence proportion ratio.

Dose-response analyzes suggested a weak non-linear association between SSB and body weight (*P* non-linear = 0.04), but not with waist circumference (*P* non-linear = 0.24), overweight/obesity incidence (*P* non-linear = 0.27) or abdominal obesity incidence (*P* non-linear = 0.70; Figures 1, 2). Each increase in SSB serving/day was associated with a +0.03 (SE 0.01) kg/year increase in weight change and a +0.05 (SE 0.01) cm/year increase in waist circumference change after adjusting for height and baseline weight or waist circumference (model 2). Further adjustment for lifestyle variables and total energy intake slightly attenuated this association, +0.02 (SE 0.01) kg/year and 0.04 (SE 0.01) cm/year (Table 2). Similarly, each SSB serving/day increase was associated with a 5% increase in incidence of overweight/obesity (IPR 1.05, 95%CI: 1.03–1.08) and a 5% increase in abdominal obesity incidence (IPR: 1.05, 95%CI: 1.02–1.07; model 2). After adjustment for dietary and lifestyle variables, these associations were attenuated to 3% (IPR: 1.03, 95%CI: 1.00–1.06) and 2% (IPR: 1.02, 95%CI: 0.99–1.05), respectively (Table 2).

**Table 2:** Linear associations between sugar-sweetened beverages, low/no-calorie beverages, fruit juice consumption, and weight-related outcomes in the Lifelines Cohort Study.

**Outcomes** ^**a**^	**Total *N*/cases *N***	**SSB (serving/day)**	**LNCB (serving/day)**	**Fruit Juice (serving/day)**
**Body weight change (kg/year)**	78,286			
Model 1		0.03 (0.01)	0.01 (0.01)	−0.01 (0.01)
Model 2		0.03 (0.01)	0.07 (0.01)	−0.02 (0.01)
Model 3		0.02 (0.01)	0.06 (0.01)	−0.02 (0.01)
Model 4		0.02 (0.01)	0.06 (0.01)	−0.02 (0.01)
**Waist circumference change (cm/year)**	78,286			
Model 1		0.03 (0.01)	0.02 (0.01)	−0.00 (0.01)
Model 2		0.05 (0.01)	0.13 (0.01)	−0.00 (0.01)
Model 3		0.03 (0.01)	0.11 (0.01)	−0.01 (0.01)
Model 4		0.04 (0.01)	0.11 (0.01)	−0.00 (0.01)
**Overweight/obesity incidence (IPR, 95%CI)** ^**b**^	35,202/4,884			
Model 1		1.02 (0.99–1.05)	1.20 (1.17–1.22)	0.98 (0.94–1.03)
Model 2		1.05 (1.03–1.08)	1.10 (1.07–1.13)	1.00 (0.96–1.04)
Model 3		1.02 (0.99–1.05)	1.08 (1.05–1.11)	0.99 (0.95–1.03)
Model 4		1.03 (1.00–1.06)	1.08 (1.06–1.11)	1.00 (0.96–1.04)
**Abdominal obesity incidence (IPR, 95%CI)** ^**c**^	31,292/6,896			
Model 1		1.04 (1.01–1.07)	1.12 (1.10–1.14)	1.03 (1.00–1.06)
Model 2		1.05 (1.02–1.07)	1.06 (1.04–1.09)	1.03 (0.99–1.06)
Model 3		1.01 (0.99–1.04)	1.05 (1.02–1.07)	1.03 (0.99–1.06)
Model 4		1.02 (0.99–1.05)	1.05 (1.02–1.07)	1.03 (1.00–1.07)

a *Results given are β (SE) for body weight and waist circumference changes or as IPR (95%CI) for overweight/obesity and abdominal obesity incidences*.

b *In participants with normal BMI (<25 kg/m^2^) at baseline*.

c *In participants with normal waist circumference (≤94 cm for men and ≤80 cm for women) at baseline*.

*Model 1: adjusted for age and sex, Model 2: model 1 + height and baseline weight (or baseline BMI for overweight/obesity incidence models) or baseline waist circumference (for models with waist circumference or abdominal obesity as outcome). Model 3: model 2 + education (categorical), physical activity (continuous), sedentary behavior (continuous), smoking (categorical), alcohol intake (categorical) + intakes of fruits, vegetables, legumes, nuts, meat, dairy, sugary foods, potatoes, fats, grains, coffee and tea (g/day) + LNCB and Fruit juice (if model SSB and vice versa) + history of diseases. Model 4: model 3 + total energy intake (kcal/day)*.

*BMI, body mass index; SSB, sugar-sweetened beverages; LNCB, low/no-calorie beverages; CVD, cardiovascular diseases; IPR, incidence proportion ratio*.

Dose-response analyzes showed linear associations between LNCB consumption and weight and waist circumference (*P* = 0.75 and *P* = 0.07; Figure 1), and a non-linear association between LNCB and overweight/obesity incidence (*P* non-linear = 0.04; Figure 2). LNCB consumption was associated with neither changes in body weight nor waist circumference after adjusting for age and sex (model 1; Table 2). However, in fully adjusted models, each increase of one serving/day LNCB was associated with a +0.06 (SE 0.01) kg/year body weight change and a +0.11 (SE 0.01) cm/year waist circumference change. Moreover, after adjustment for age and sex, each LNCB serving/day increase was associated with a 20% increase in incidence of overweight/obesity (IPR: 1.20, 95%CI: 1.17–1.22) and a 12% higher incidence of abdominal obesity (IPR: 1.12, 95%CI: 1.04–1.09; model 1; Table 2), which attenuated to an 8% (IPR: 1.08, 95%CI: 1.06–1.11) and a 5% (IPR: 1.05, 95%CI: 1.02–1.07) increase after full adjustment (model 4). The non-linear association between LNCB and overweight/obesity incidence showed a steeper increase in risk <1 serving/day intake and a more gradual increase at higher levels (Figure 2 and Table 3).

**Table 3:** Adjusted associations between sugar-sweetened beverages, low/no-calorie beverages, fruit juice consumption, and weight-related outcomes categorized by intake levels.

**Outcomes** ^**a**^		**SSB**				**LNCB**				**Fruit Juice**			
		**None**	**≤1 serving/day**	**1–2 servings/day**	**>2** **servings/day**	**None**	**≤1 serving/day**	**1–2 servings/day**	**>2** **servings/day**	**None**	**≤1 serving/day**	**1–2 servings/day**	**>2** **servings/day**
	***N*** **total/cases**	29,637	36,967	8,134	3,548	33,938	33,497	7,697	3,154	18,220	51,831	6,945	1,290
Body weight change (kg/year)	78,286	ref	−0.04 (0.01)	−0.03 (0.02)	0.04 (0.03)	ref	0.03 (0.01)	0.09 (0.02)	0.20 (0.03)	ref	−0.07 (0.01)	−0.07 (0.01)	−0.09 (0.05)
Waist circumference change (cm/year)	78,286	ref	−0.03 (0.02)	−0.01 (0.03)	0.09 (0.04)	ref	0.08 (0.02)	0.20 (0.03)	0.40 (0.04)	ref	−0.06 (0.02)	−0.03 (0.03)	−0.02 (0.03)
Overweight/ obesity^b^	35,202/4,884	ref	0.94 (0.88–1.00)	0.92 (0.83–1.01)	1.16 (1.04–1.28)	ref	1.06 (1.01–1.11)	1.18 (1.10–1.26)	1.26 (1.13–1.38)	ref	0.89 (0.83–0.95)	0.94 (0.84–1.03)	1.00 (0.82–1.18)
Abdominal obesity ^c^	31,292/6,896	ref	0.99 (0.95–1.04)	1.01 (0.93–1.08)	1.06 (0.95–1.16)	ref	1.06 (1.02–1.10)	1.12 (1.05–1.19)	1.13 (1.02–1.25)	ref	0.99 (0.94–1.04)	1.05 (0.97–1.13)	1.04 (0.90–1.21)

a *Results given are β (SE) for body weight and waist circumference changes or as IPR (95%CI) for overweight/obesity and abdominal obesity incidences*.

b *For overweight/obesity incidence in each intake category n = 11,858; n = 177,740; n = 3,861 and n = 1,743 for SSB; n = 16,935; n = 14,745; n = 2,658 and n = 864 for LNCB, and n = 7,124; n = 24,230; n = 3,255 and n = 593 for Fruit Juice*.

c *For abdominal obesity incidence in each intake category n = 9,631, n = 16,187; n = 3,731 and n = 1,743 for SSB; n = 14,927, n = 13,042, n = 2,436 and n = 887 for LNCB and n = 6,128; n = 24,446; n = 3,113; and n = 605 for Fruit Juice*.

*All models were adjusted by age, sex, height, and baseline weight (or baseline BMI for overweight/obesity) or baseline waist circumference (for models with waist circumference and abdominal obesity models as outcome), education (categorical), physical activity (continuous), sedentary behavior (continuous), smoking (categorical), alcohol intake (categorical), intakes of fruits, vegetables, legumes, nuts, meat, dairy, sugary foods, potatoes, fats, grains, coffee and tea (g/day), LNCB and Fruit juice (if model SSB and vice versa), history of diseases (diabetes, CVD, hypertension, and hypercholesterolemia) and total energy intake (kcal/day) (model 4)*.

*BMI, body mass index; SSB, sugar-sweetened beverages; LNCB, low/no-calorie beverage; CVD, cardiovascular diseases; IPR, incidence proportion ratio*.

Dose-response analyzes also indicated non-linearity for the associations between fruit juice with weight and waist circumference (*P* **<** 0.001 and *P* = 0.01, respectively; Figure 1). Below an intake of ~1 serving fruit juice/day, body weight and waist circumference decreased, while no associations were observed above this threshold (Table 3). Accordingly, a J-shaped association was present for the association between fruit juice intake and overweight/obesity incidence (*P* non-linear = 0.02; Figure 2). Compared to non-users, participants consuming ≤1 serving/day had an 11% reduced overweight/obesity risk (IPR: 0.89, 95%CI: 0.83–0.95) while no association was observed at higher intake levels (Table 3). A weak linear association was observed for fruit juice and abdominal obesity incidence (IPR: 1.03, 95%CI 1.00–1.06; *P* non-linear = 0.49; Figure 2 and Table 3).

Adjusting for or excluding participants with “desire to lose weight” or participants with self-reported health conditions at baseline (i.e. type 2 diabetes, CVD, hypertension, and hypercholesterolemia) did not substantially alter the associations for any beverage (Supplementary Tables 2, 3).

**Figure 3:**
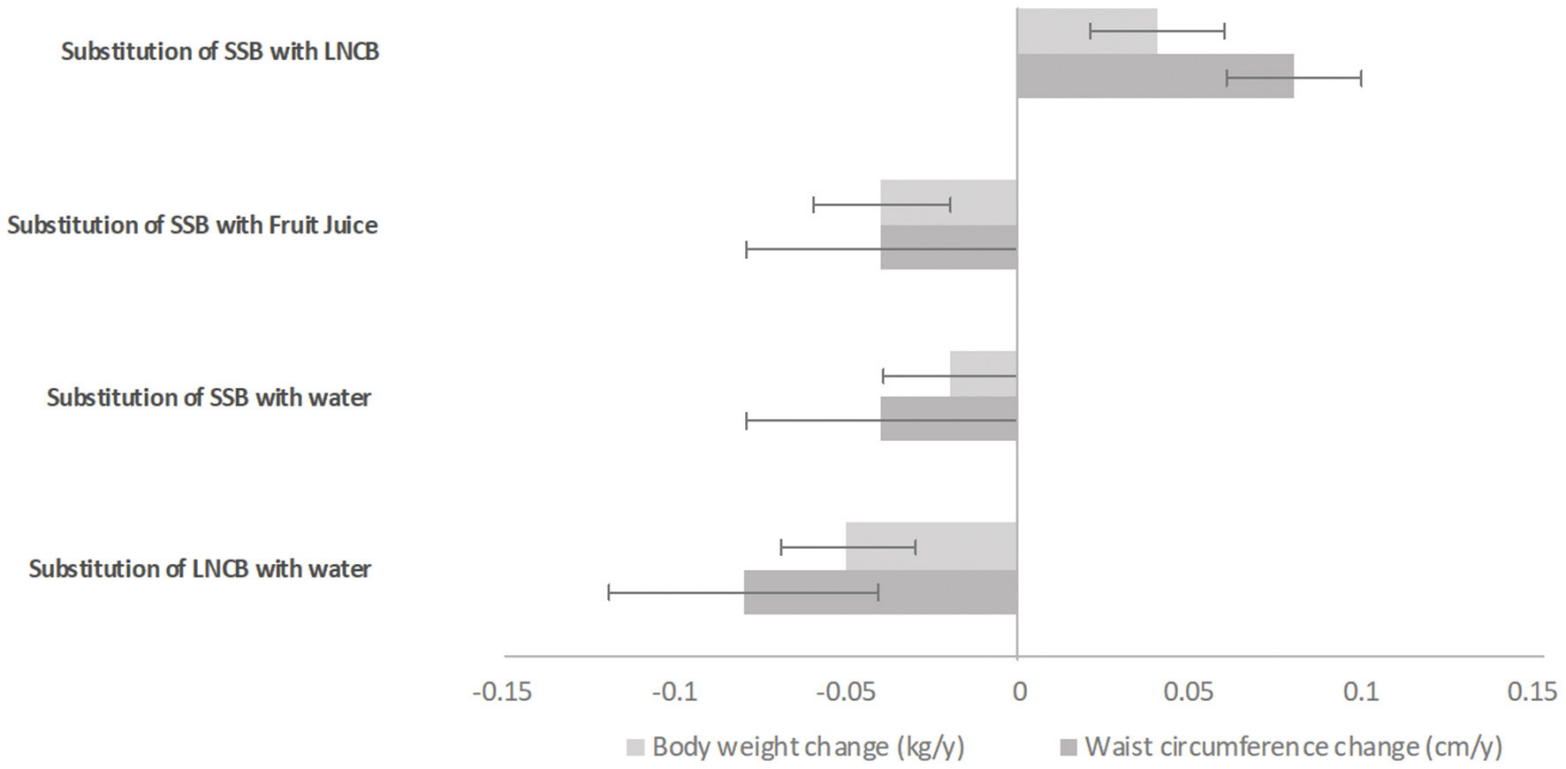
Adjusted substitution associations of one serving of beverage with another with body weight change (kg/year) and waist circumference change (cm/year) in the Lifelines Cohorts Study; Models were adjusted for age, sex, height, baseline weight or baseline waist circumference (for models with waist circumference as outcome), education, alcohol intake, smoking, physical activity, all dietary factors and history of diseases. Error bars represent the 95% CIs. SSB, sugar-sweetened beverages; LNCB, low/no-calorie beverages.

**Figure 4:**
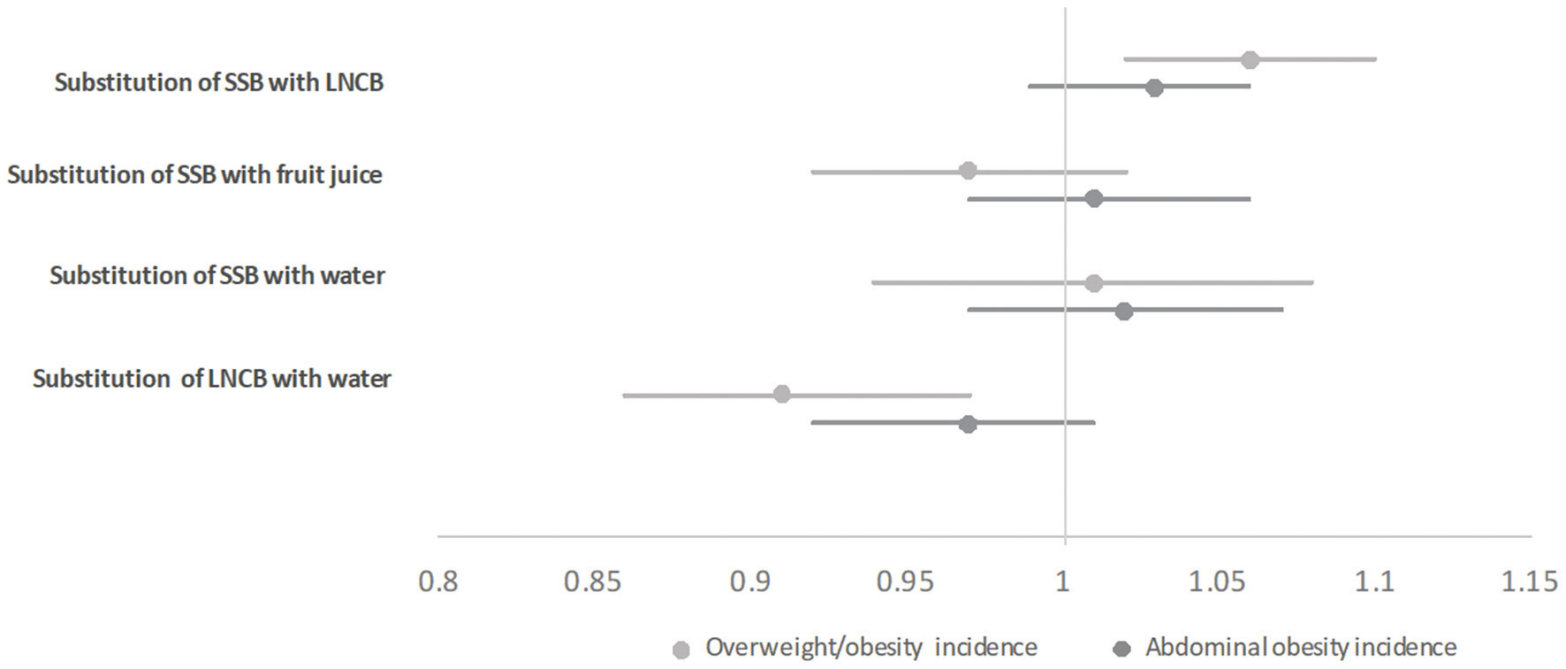
Adjusted substitution associations of one serving of beverage with another with overweight/obesity incidence (IPR, 95%CI) and abdominal obesity incidence (IPR, 95%CI) in participants with normal values at baseline (i.e., <25 kg/m^2^ for overweight/obesity and ≤94 cm in men and ≤80 cm in women for abdominal obesity) in the Lifelines Cohort Study; Models were adjusted for age, sex, baseline BMI or baseline waist circumference and height (for models with abdominal obesity as outcome), education, alcohol intake, smoking, physical activity, all dietary factors, and history of diseases; SSB, sugar-sweetened beverages; LNCB, low/no-calorie beverage; IPR, incidence proportion ratio.

Substitution analyzes are shown in Figures 3, 4. Replacing one serving SSB with an equal amount of water or fruit juice was associated with decreased weight (−0.02 kg/year, SE 0.01 and −0.04 kg/year, SE 0.01, respectively) and waist circumference (−0.04 cm/year, SE 0.02; for both beverages) (Figure 3). Substituting LNCB with water also showed an inverse association with weight (−0.05 kg/year, SE 0.01) and waist circumference (−0.08 cm/year, SE 0.01). In contrast, replacing SSB with LNCB was positively associated with increased weight (+0.04 kg/year, SE 0.01) and waist circumference (+0.08 cm/year, SE 0.01). Only the substitution of one serving SSB with an equal amount of LNCB was associated with higher overweight/obesity incidence (IPR: 1.06; 95%CI: 1.02–1.10) while the substitution of one serving LNCB with one serving water was associated with reduced overweight/obesity incidence (IPR: 0.91; 95%CI: 0.86–0.97) (Figure 4). None of the other substitution analyzes related to overweight or abdominal obesity incidences showed any association.

Stratified analyzes are included as Supplementary Tables 4–6. Analyzes stratified by BMI category showed an interaction for SSB consumption and body weight change (*P* interaction < 0.001; Supplementary Table 4). In participants with normal BMI (< 25 kg/m^2^), each increase in SSB serving/day was associated with a +0.04 (SE 0.01) kg/year change in body weight while in participants with overweight/obesity no association was observed (0.00 kg/year, SE 0.01). Moreover, the impact of substituting one serving SSB with an equal amount of LNCB was more pronounced in overweight/obese participants (+0.06 kg/year, SE 0.01) compared to participants with normal BMI (+0.02 kg/year; SE 0.01, *P* interaction **<** 0.001; Supplementary Table 6). Stratification by BMI also showed an interaction for fruit juice and body weight (*P* interaction **<** 0.01) with a stronger inverse association among participants with overweight/obesity (Supplementary Tables 4, 5). Additional stratification by sex indicated that the association of LNCB with abdominal obesity was slightly more pronounced in men (IPR: 1.06, 95%CI: 1.03–1.10) than in women (IPR: 1.03, 95%CI: 1.00–1.06, *P*-interaction = 0.04; Supplementary Table 4) with a similar observation for the substitution analysis (Supplementary Table 6). In contrast, the beneficial association observed between fruit juice and body weight change at moderate doses was stronger in women than in men (*P* interaction = 0.03). We did not observe any other substantial evidence of effect modification with BMI, sex, or age.

## Discussion

In our study among 78,286 Dutch adults, habitual intakes of SSB and LNCB were linearly associated with most weight outcomes. A J-shaped association was observed for fruit juice showing a beneficial consumption below ~1 serving/day for all outcomes except abdominal obesity incidence. In addition, the theoretical substitution of SSB with LNCB was associated with an increase in weight and waist circumference. Replacing one serving SSB with an equal amount of fruit juice was associated with decreases in weight and weight circumference.

The positive associations observed between SSB and weight outcomes in our study are generally in line with earlier studies (4, 16, 17). A meta-analysis including 174,252 adults, mostly from the US, showed that each daily serving (= 250 ml) increase in SSB was associated with a 0.22 kg (95%CI: 0.50–1.20) higher weight gain over 1 year (4). Furthermore, the positive associations between SSB consumption and overweight/obesity incidence align with recent meta-analyzes by Schlesinger et al. (16) and Qin et al. (17), which reported an increased overweight/obesity and obesity risk with each additional SSB serving, i.e., 5% (RR: 1.05, 95%CI:1.00–1.11) (16) and 12% (RR: 1.12, 95%CI:1.05–1.19), respectively (17). However, it needs to be emphasized that our results are rather modest compared to these – predominantly US - studies, which may be explained by lower SSB intake levels in our population (4, 16, 17) and a relatively short follow-up time of ~4 years in our study. More consistent results may be observed once the follow-up time exceeds 5 years (34).

We also observed positive associations between LNCB consumption and body weight outcomes; stratifying the data for those with normal vs. overweight showed similar results. Moreover, substitution analyzes, showed that the replacement of SSB by LNCB was adversely associated with weight and waist circumference changes. Previous meta-analyzes of prospective studies on Low/No calorie sweeteners and weight outcomes generally report either no association or positive associations (9, 12, 13, 17, 35). To illustrate, a recent meta-analysis of prospective studies reported a 21% (RR: 1.21) increased risk of obesity for each 250 ml LNCB increment (17). The latest WHO report also acknowledges observed positive prospective associations of LNCB consumption with incidence obesity and BMI, but not with other adiposity measures (36). However, meta-analyzes of RCTs do not support these observational findings and generally report a beneficial impact of LNCB on body weight measures (9–11, 13, 36, 37). Conflicting findings between observational studies and RCTs might be due to differences in design and follow-up time where potential reverse causation or residual confounding may explain adverse findings in observational studies. It may be that overweight participants consume LNCB instead of SSB to manage their weight, while their overall weight management strategy is not sufficiently effective. This phenomenon of reverse causality may explain why the adverse association of replacing SSB with LNCB in our study was slightly stronger in the higher BMI category and why replacing LNCB – but not water – for SSB showed an adverse association with incidence overweight/obesity. To date, only a few other prospective studies have investigated the theoretical substitution of different beverages (20–22). In our study, substituting both SSB and LNCB with water was associated with less weight and waist circumference gain. Other studies have found similar beneficial results for the replacement of SSB with water and associations with weight change (20) or incidence obesity (21). However, substituting LNCB with water was not associated with any adiposity measures in other prospective analyzes (21, 22).

Interestingly, we found J-shaped associations between fruit juice and weight, waist circumference, and incidence of overweight/obesity during follow-up. Non-linear continuous dose-response associations between fruit juice consumption and weight-related outcomes have not been reported before. However, our results are in line with previous findings for CVD risk (18, 19). Khan et al. (19) observed a non-linear J-shaped curve with a beneficial association between 100% fruit juice and CVD incidence at moderate doses (~150 ml) but no association at higher doses. D'Elia et al. (18) reported similar results in prospective studies of 100% fruit juice with CVD incidence. However, the borderline linear association observed with abdominal obesity incidence suggests that the consumption of fruit juice, even at moderate intake, might still be recommended against. Thus, further research on the potential beneficial effect of fruit juice is warranted.

Mechanically, the adverse association between SSB and increased body weight can be supported by several biological mechanisms (28, 29). The high-calorie content of SSBs and the lack of energy compensation can lead to a disturbed energy balance and thus weight gain. SSBs also contain rapidly absorbable carbohydrates that affect insulin secretion and blood glucose and possibly later insulin resistance (30). In contrast, biological mechanisms for the association between LNCB and weight are unclear. Factors other than energy intake may explain the adverse associations found with habitual LNCB and future weight gain. LNCB has been suggested to indirectly affect intestinal glucose absorption, appetite, and hormone dysregulation through activation of sweet taste receptors (38, 39). Other potential mechanisms include altered gut microbiota leading to glucose intolerance and insulin resistance (38, 39). Nevertheless, evidence for these mechanisms in human RCTs is limited and was not demonstrated by other studies when compared to water or unsweetened products (9, 40–42) or when used as a control in RCTs of SSB (43, 44). In contrast to SSB or LNCB, Fruit juices contain health-promoting nutrients such as antioxidants (i.e., polyphenols) and other bioactive substances (i.e., vitamins and minerals) (45). These nutrients could explain the benefits observed with moderate consumption of fruit juice on certain health outcomes as they may play a role in lowering oxidative stress, inflammation, and improving glucose metabolism (46, 47). After a certain level, these benefits may be counterbalanced by the sugar and calorie content of fruit juice leading to a detrimental effect on body weight measures through similar mechanisms as SSB. However, such a phenomenon remains a hypothesis (46).

An important strength of our study is the large sample size allowing for well-powered stratification and adjustment for multiple covariates. Our study is one of the largest studies on the association and replacement of sweet beverages conducted in European adults so far. In addition, we used prospective measures of body weight and waist circumference, rather than self-reported anthropometrics. Furthermore, we included a variety of outcome measures, i.e., continuous waist circumference change, obesity, and abdominal obesity incidence. And last but not least, we evaluated both measures of continuous and dichotomous weight and waist-related outcomes together with the exploration of non-linear dose-response and substitution associations. A limitation of this study is that habitual dietary intake is only assessed at baseline. Dietary assessment at multiple time points might have provided more insight into whether the adverse associations observed in observational studies are caused by the beverage itself or other associated behaviors. Second, we were not able to distinguish between different types of fruit juice and different types of LNCB, and, therefore, we were not able to investigate potential differential effects of consumed sweeteners on weight gain. For example, a recent study demonstrated increased weight gain and hunger with saccharin intake but no change in energy intake, indicating that mechanisms other than energy intake might be implicated for this specific sweetener (48). With the use of different blends of sweeteners on the market, it is particularly relevant to further investigate specific effects in the future. Third, our questionnaire included 100% fruit juice along with other fruit drinks, which limits comparison with other studies that used specifically 100% fruit juice. However, according to the last consumption survey in the Netherlands, pasteurized orange, apple, and mixed juices composed 90% of the total fruit juice consumption in the Netherlands in the same time period as our study (49). Thus, we assume that the fruit juices consumed in this study were mostly 100% fruit juice.

To conclude, our study indicates that habitual consumption of both SSB and LNCB may adversely affect weight-related outcomes. In contrast, consumption of moderate amounts of fruit juice (<150 ml) may be beneficial with respect to body weight and waist circumference.

## Data Availability Statement

The data analyzed in this study is subject to the following licenses/restrictions: Data described in the manuscript, codebook, and analytic code will be made available upon request pending application, payment, and SWEET consortium agreement. Requests to access these datasets should be directed at: onderzoek@lifelines.nl.

## Ethics Statement

The studies involving human participants were reviewed and approved by The Medical Ethical Review Committee of the University Medical Center in Groningen. The patients/participants provided their written informed consent to participate in this study.

## Author Contributions

JHar, JHal, and AR are coordinators of the SWEET project and together with EF initiated the research question. MB and NN prepared the data for analyzes. MB, EB-B, and EF analyzed the data and drafted the manuscript. All authors critically revised the manuscript for important intellectual content and approved of the final version to be published.

## Funding

This EU-project under the acronym SWEET has received funding from the European Union's Horizon 2020 research and innovation programme, grant agreement No. 774293.

## Conflict of Interest

JHal is on the International Sweeteners and Mars Scientific Advisory Boards and together with JHar and AR have received honorariums from the international Sweeteners association. JHal and JHar are also conducting the SWITCH trial funded by the American Beverage Association. AR has received an honorarium from Unilever. EF has received an unrestricted grant from Friesland Campina and European Beer Institute and conducted a study on added sugar and individual sugars partly funded by Kenniscentrum Suiker en Gezondheid. MB, NN, and EB-B report no conflict of interest. The funding sponsors had no role in the design of the study; in the collection, analyzes, interpretation of data, writing of the manuscript, and nor in the decision to publish the results.

## Publisher's Note

All claims expressed in this article are solely those of the authors and do not necessarily represent those of their affiliated organizations, or those of the publisher, the editors and the reviewers. Any product that may be evaluated in this article, or claim that may be made by its manufacturer, is not guaranteed or endorsed by the publisher.
