# Supplementary material for: Dose-Response and Substitution Analyzes of Sweet Beverage Consumption and Body Weight in Dutch Adults: The Lifelines Cohort Study
Source: Front Nutr. 2022 Jun 24;9:889042. doi: 10.3389/fnut.2022.889042 (PMC9272075; doi:10.3389/fnut.2022.889042)
Supplement: Supplementary file 1 [file Table_1.docx]

***Supplementary material***

The Lifelines Cohort Study

**N initial = 152,728**

N = 6,259 Excluded due to missing covariates

n = 335 participants had missing data for education

n = 5,788 participants had missing data for physical activity

n = 190 participants had missing data for smoking status

**N = 128,612**

**N = 84,545**

Included in analysis

**N = 78,286**

N = 24,116 excluded due to missing or unreliable dietary data (men with energy intakes <800 or >4000 kcal/d or women with energy intakes <500 or >3500 kcal/d)

N = 44,067 excluded due to missing variable for yearly weight change and waist circumference change

Supplemental Figure 1. Flow chart of the Lifelines Cohort participants

Supplementary Figure 1 Flow Chart of the Lifelines Cohort Study

Supplementary Table 1. Baseline characteristics by beverages intake in the Lifelines Cohort Study

| Characteristics ^a^ | SSB | | | LNCB | | | Fruit juice | | |
| --- | --- | --- | --- | --- | --- | --- | --- | --- | --- |
|  | 0  serving | ≤1 serving  /d | >1 serving/d | 0  serving | ≤1 serving  /d | >1 serving/d | 0  serving | ≤1 serving  /d | >1 serving/d |
| N | 29,637 | 36,967 | 11,682 | 33,938 | 33,497 | 10,851 | 18,220 | 51,831 | 8,235 |
| Demographic and SES |  |  |  |  |  |  |  |  |  |
| Women (%) | 20637 (69.6) | 20662 (55.9) | 5364 (45.9) | 19619 (57.8) | 20590 (61.5) | 6454 (59.5) | 11173 (61.3) | 31200 (60.2) | 4290 (52.1) |
| Age, years | 50.5 (11.6) | 44.5 (12.4) | 38.3 (11.5) | 47.0 (13.0) | 45.7 (12.6) | 42.7 (11.5) | 48.6 (12.6) | 45.6 (12.6) | 41.1 (12.0) |
| Education, n (%)  low  intermediate  high | 1405 (4.7)  19002 (64.1)  9230 (31.1) | 1240 (3.4)  23321(63.1)  12406 (33.6) | 432 (3.7)  8367 (71.6)  2883 (24.7) | 1487 (4.4)  21885 (64.5)  10566 (31.1) | 1205 (3.6)  21405 ( 63.9)  10887 (32.5) | 385 (3.5)  7400 (68.2)  3066 (28.3) | 970 (5.3)  12937 (71.0)  4313 (23.7) | 1827 (3.5)  32337 (62.4)  17667 (34.1) | 280 (3.4)  5416 (65.8)  2539 (30.8) |
| Anthropometrics |  |  |  |  |  |  |  |  |  |
| Body weight, kg | 79.2 (15.0) | 79.3 (14.6) | 81.2 (15.7) | 77.9 (14.4) | 79.5 (14.6) | 84.6 (16.8) | 80.5 (15.4) | 79.0 (14.7) | 80.6 (15.7) |
| Waist circumference, cm | 90.8 (12.4) | 89.5 (11.8) | 90.1 (12.6) | 89.1 (11.9) | 90.0 (11.9) | 93.5 (13.5) | 91.7 (12.6) | 89.5 (11.9) | 89.9 (12.4) |
| BMI, kg/m² | 26.5 (4.3) | 25.7 (4.0) | 25.7 (4.3) | 25.5 (4.0) | 26.0 (4.1) | 27.5 (4.8) | 26.6 (4.4) | 25.8 (4.1) | 25.8 (4.3) |
| Categories BMI  normal  overweight  obese | 11858 (40.0)  12426 (41.9)  5353 (18.1) | 17740 (48.0)  14,617 (39.5)  4,610 (12.5) | 5604 (48.0)  4374 (37.4)  1704 (14.6) | 16935 ( 49.9)  13025 ( 38.4)  3978 ( 11.7) | 14745 ( 44.0)  13869 ( 41.4)  4883 (14.6) | 3522 (32.5)  4523 (41.7)  2806 (25.9) | 7124(39.1)  7721 (42.4)  3375 (18.5) | 24230 (46.7)  20534 (39.6)  7067 (13.6) | 3848 (46.8)  3162 (38.4)  1225 (14.9) |
| Desire to lose weight | 18548 (62.7) | 19992 (54.2) | 5871 (50.3) | 16922 (49.9) | 19900 (59.5) | 7589 (70.1) | 10681 (58.7) | 29331 (56.7) | 4399 (53.5) |
| Lifestyle |  |  |  |  |  |  |  |  |  |
| Physical activity (METs)  Intense  Moderate  Sedentary(min/week) | 0 [0, 558]  1780[855, 3143]  1050 [630, 1260] | 0 [0,630]  1649[816, 2904]  840 [630, 1260] | 0 [0,600]  1438 [630, 2670]  1050 [840, 1260] | 0 [0, 540]  1647 [775.5, 2972.0]  840 [630, 1260] | 0 [0, 630]  1722[854, 2979]  840 [630, 1260] | 0 [0, 630]  1506 [700, 2800]  1050 [840, 1470] | 0 [0, 456]  1628 [735, 3012]  1050 [840,1470] | 0 [0, 630]  1680 [836, 2940]  840 [630,1260] | 0 [0, 720]  1628 [753, 2976]  840 [630,1260] |
| Smoking  never  former  current | 12095 (40.8)  12902 (43.5)  4640 (15.7) | 18659 (50.5)  11790 (31.9)  6518 (17.6) | 577 (48.9)  2684 (23.0)  3291 (28.2) | 15345 (45.2)  12055 (35.5)  6538 (19.3) | 16201 (48.4)  11737 (35.0)  5559 (16.6) | 4915 (45.3)  3584 (33.0)  2352 (21.7) | 7171 (39.4)  7459 (40.2)  3590 (19.7) | 25129 (48.5)  17695 (34.1)  9007 (17.4) | 4161 (50.5)  2222 (27.0)  1852 (22.5) |
| Alcohol intake, n (%)  No alcohol  Medium (0-≤10g)  High (10g-≤20g)  Very high (>20g) | 1045 (3.5)  20561 (69.4)  5961 (20.1)  2070 (7.0) | 587 (1.6)  27024 (73.1)  6970 (18.9)  2386 (6.5) | 287 (2.5)  8303 (71.1)  2101 (18.0)  991 (8.5) | 1001 (2.9)  23605 (69.6)  6672 (19.6)  2660 (7.8) | 612 (1.8)  24544 (73.3)  6333 (18.9)  2008 (6.0) | 306 (2.8)  7739 (71.3)  2027 (18.7)  779 (7.2) | 818 (4.5)  12397 (68.0)  3489 (19.1)  1516 (8.3) | 909 (1.8)  37611 (72.6)  9984 (19.3)  3327 (6.4) | 192 (2.3)  5880 (71.4)  1559 (18.9)  604 (7.3) |

**Supplementary Table 1.** cont.

| **Characteristics** |  | **SSB** |  | **LNCB** |  |  | **Fruit juice** |  |  |
| --- | --- | --- | --- | --- | --- | --- | --- | --- | --- |
|  | 0  serving | <1 serving  /d | >1 servings/d | 0  serving | <1 serving  /d | >1 servings/d | 0  serving | <1 serving  /d | >1 servings/d |
| Dietary intake |  |  |  |  |  |  |  |  |  |
| Total energy, g/d | 1782 [1486, 2129] | 2043 [1719, 2425] | 2317 [1935, 2750] | 1992 [1643, 2408] | 1957 [1632, 2340] | 1996 [1652, 2408] | 1829 [1505, 2230] | 1989 [1662, 2373] | 2242 [1866, 2664] |
| Fruit, g/d | 152.4 [76.2, 220.2] | 110.1 [42.3, 220.2] | 76.2 [21.1, 152.4] | 110.1 [42.3, 220.2] | 110.1 [76.2, 220.2] | 84.6 [42.3, 220.2] | 110.1 [42.3, 220.2] | 110.1 [42.3, 220.2] | 84.6 [42.3, 220.2] |
| Grains and cereals, g/d | 166.3 [125.1, 213.5] | 187.5 [145.5, 239.7] | 188.4 [143.0, 245.3] | 180.0 [136.3, 233.2] | 180.2 [138.5, 230.1] | 176.2 [133.3, 226.8] | 169.1 [125.8, 220.1] | 181.5 [139.6, 232.3] | 189.9 [145.0, 244.3] |
| Potatoes, g/d | 73.6 [46.7, 104.1] | 92.6 [58.7, 121.1] | 96.2 [63.3, 128.4] | 88.1 [53.8, 118.4] | 88.1 [55.8, 111.2] | 85.6 [53.6, 111.2] | 88.1 [52.0, 118.4] | 88.1 [55.7, 111.2] | 88.1 [57.0, 121.1] |
| Fats, g/d | 19.8 [10.1, 28.9] | 23.7 [13.6, 33.0] | 24.3 [12.9, 35.0] | 22.7 [12.2, 32.5] | 22.3 [12.5, 31.4] | 21.0 [10.5, 31.3] | 21.4 [11.1, 31.5] | 22.4 [12.5, 31.8] | 23.4 [12.5, 33.6] |
| Vegetables, g/d | 110.5 [74.5, 152.0] | 108.0 [63.5, 114.4] | 75.6 [61.9, 112.8] | 108.3 [73.7, 149.4] | 108.3 [73.7, 114.4] | 105.9 [62.4, 114.4] | 108.3 [73.7, 149.1] | 108.3 [73.7, 148.7] | 104.1 [62.4, 114.4] |
| Dairy, g/d | 272.91 [168.6, 396.7] | 275.3 [171.7, 403.7] | 237.8 [142.7, 370.6] | 263.1 [161.2, 393.5] | 278.6 [175.7, 402.5] | 252.4 [154.9, 382.3] | 264.3 [160.0, 384.4] | 271.8 [169.8, 399.5] | 258.1 [155.1, 401.8] |
| Meat, g/d | 71.8 [46.2, 95.2] | 76.9 [60.7, 99.3] | 85.4 [64.7, 107.0] | 74.3 [50.0, 97.9] | 75.6 [58.0, 97.9] | 82.6 [63.5, 103.6] | 75.5 [54.2, 98.8] | 75.6 [56.0, 98.3] | 78.2 [60.7, 101.6] |
| Sugary foods, g/d | 52.5 [31.2, 80.5] | 67.8 [43.8, 99.0] | 76.0 [47.4, 113.5] | 61.2 [36.8, 93.3] | 63.8 [40.7, 94.1] | 64.6 [39.5, 98.5] | 54.4 [31.4, 85.5] | 64.6 [41.0, 95.7] | 69.2 [43.5, 104.6] |
| Legumes, g/d | 11.1 [0.0, 28.8] | 13.3 [0.0, 30.2] | 11.0 [0.0, 27.6] | 11.1 [0.0, 29.5] | 13.3 [0.0, 29.5] | 11.0 [0.0, 27.6] | 11.0 [0.0, 27.1] | 11.8 [0.0, 29.5] | 11.8 [0.0, 32.9] |
| Nuts, g/d | 7.3 [2.8, 15.9] | 8.4 [3.5, 16.7] | 8.3 [2.8, 16.6] | 8.2 [2.9, 17.1] | 8.3 [3.4, 15.9] | 8.1 [2.9, 16.5] | 6.9 [2.1, 15.1] | 8.3 [3.4, 16.5] | 8.8 [3.4, 18.4] |
| Coffee, g/d | 464.5 [232.3, 580.6] | 464.5 [232.3, 580.6] | 348.4 [116.1, 580.6] | 464.5 [232.3, 580.6] | 464.5 [232.3, 580.6] | 464.5 [232.3, 580.6] | 464.5 [232.3, 580.6] | 464.5 [232.3, 580.6] | 348.4 [160.8, 580.6] |
| Tea, g/d | 232.3 [80.4, 464.5] | 232.3 [44.6, 348.4] | 116.1 [17.9, 241.1] | 232.3 [44.6, 348.4] | 232.3 [80.4, 348.4] | 160.8 [22.3, 348.4] | 232.3 [35.8, 348.4] | 232.3 [80.4, 348.4] | 160.8 [35.8, 348.4] |
| Sweet beverages |  |  |  |  |  |  |  |  |  |
| SSB, servings/d | 0.0 [0.0, 0.0] | 0.3 [0.1, 0.6] | 1.8 [1.3, 2.5] | 0.1 [0.0, 0.7] | 0.1 [0.0, 0.4] | 0.0 [0.0, 0.8] | 0.0 [0.0, 0.3] | 0.1 [0.0, 0.6] | 0.4 [0.0, 1.3] |
| LNCB, servings/d  Fruit Juice, servings/d | 0.1 [0.0, 0.7]  0.1 [0.0, 0.4] | 0.1 [0.0, 0.4]  0.2 [0.1, 0.7] | 0.0 [0.0, 0.6]  0.3 [0.1, 0.9] | 0.0 [0.0, 0.0]  0.1 [0.0, 0.6] | 0.3 [0.1, 0.6]  0.2 [0.1, 0.6] | 1.9 [1.3, 2.5]  0.2 [0.0, 0.7] | 0.0 [0.0, 0.6]  0.0 [0.0, 0.0] | 0.1 [0.0, 0.5]  0.3 [0.1, 0.6] | 0.1 [0.0, 0.7]  1.8 [1.3, 1.9] |
| History of diseases |  |  |  |  |  |  |  |  |  |
| Type 2 diabetes | 1309 (4.4) | 471 (1.3) | 73 (0.6) | 601 (1.6) | 780 (2.3) | 472 (4.3) | 789 (4.3) | 967 (1.9) | 97 (1.2) |
| CVD | 859 (2.9) | 734 (2.0) | 212 (1.8) | 828 (2.4) | 735 (2.2) | 242 (2.2) | 587 (3.2) | 1073 (2.1) | 145 (1.8) |
| Hypertension | 8388 (28.3) | 7301 (19.8) | 1810 (15.5) | 7210 (21.2) | 7682 (22.9) | 2607 (24.0) | 4732 (26.0) | 11319 (21.8) | 1448 (17.6) |
| Hypercholesterolemia | 5492 (18.5) | 4542 (12.3) | 1036 (8.9) | 4789 (14.1) | 4685 (14.0) | 1596 (14.7) | 3235 (17.8) | 6977 (13.5) | 858 (10.4) |
| Outcomes |  |  |  |  |  |  |  |  |  |
| Body Weight change (kg/y) | -0.01 (1.67) | 0.01 (1.47) | 0.14 (1.68) | 0.02 (1.49) | 0.01 (1.54) | 0.03 (1.93) | 0.05 (1.80) | 0.01 (1.49) | 0.06 (1.63) |
| Waist circumference change (cm/y) | 0.01 (2.11) | -0.01 (1.97) | 0.07 (2.08) | 0.01 (2.01) | 0.00 (2.01) | 0.03 (2.20) | 0.04 (2.16) | 0.00 (1.99) | 0.02 (2.04) |

^a^ Mean (SD), median [25th-75^th^ percentile] or n (%).

BMI: Body mass index; SSB: Sugar-sweetened beverages; LNCB: Low/non-calorie beverages; CVD: Cardiovascular diseases.

Supplementary Table 2. Sensitivity analyzes for the linear associations of one serving per day SSB, LNCB and fruit juice with weight-related outcomes in the Lifelines Cohort Study ^a^

|  | **N** | **SSB** | **LNCB** | **Fruit Juice** |
| --- | --- | --- | --- | --- |
| **Excluding self-reported diseases** |  |  |  |  |
| Body weight change (kg/year) | 53,692 | 0.02 (0.01) | 0.05 (0.01) | -0.01 (0.01) |
| Waist circumference change (cm/year) | 53,692 | 0.03 (0.01) | 0.12 (0.01) | -0.01 (0.01) |
| Overweight/Obesity incidence | 27,756 | 1.02 (0.98-1.05) | 1.07 (1.04-1.10) | 1.01 (0.97-1.06) |
| Abdominal obesity incidence | 24,947 | 1.02 (0.99-1.05) | 1.05 (1.02-1.07) | 1.04 (1.00-1.08) |
| **Adjusted for desire to lose weight ^b^** |  |  |  |  |
| Body weight change (kg/year) | 78,152 | 0.02 (0.01) | 0.06 (0.01) | -0.02 (0.01) |
| Waist circumference change (cm/year) | 78,152 | 0.05 (0.01) | 0.11 (0.01) | 0.00 (0.01) |
| Overweight/Obesity incidence | 35,146 | 1.03 (1.00-1.06) | 1.09 (1.06-1.12) | 1.00 (0.96-1.05) |
| Abdominal obesity incidence | 31,246 | 1.03 (1.00-1.05) | 1.04 (1.01-1.06) | 1.04 (1.00-1.07) |
| **Excluding participants with**  **desire to lose weight ^b^** |  |  |  |  |
| Body weight change (kg/year) | 33,741 | 0.03 (0.01) | 0.06 (0.01) | 0.01 (0.01) |
| Waist circumference change (cm/year) | 33,741 | 0.06 (0.01) | 0.10 (0.01) | 0.01 (0.02) |
| Overweight/Obesity incidence | 24,815 | 1.01 (0.97-1.06) | 1.11 (1.07-1.16) | 1.02 (0.96-1.08) |
| Abdominal obesity incidence | 21,854 | 1.04 (1.00-1.07) | 1.05 (1.01-1.08) | 1.05 (1.01-1.10) |
| ^a^ Results given are mean (SE) or IPR (95%CI).  ^b^ 134 participants did not answer the questions regarding their desire to lose weight (i.e., wanting to lose weight).  Model adjusted for age, sex, baseline weight (or baseline BMI for overweight/obesity incidence models) or baseline waist circumference (for models with waist circumference or abdominal obesity as outcome), education (categorical), physical activity (continuous), sedentary behavior (continuous), smoking (categorical), alcohol intake (categorical), intakes of fruits, vegetables, legumes, nuts, meat, dairy, sugary foods, potatoes, fats, grains, coffee and tea (g/d) + Fruit juice and LNCB/SSB (if model SSB or LNCB), history of diseases (diabetes, CVD, hypertension and hypercholesterolemia) and total energy intake (kcal/d).  BMI: Body mass index; SSB: Sugar-sweetened beverages; LNCB: Low/non-calorie beverages; CVD: Cardiovascular diseases; IPR: Incidence proportion ratio. | | | | |

**Supplementary Table 3.** Sensitivity analyzes stratified by beverages intake categories in the Lifelines Cohort Study

|  |  | **SSB** | | | | **LNCB** | | | | **Fruit Juice** | | | |
| --- | --- | --- | --- | --- | --- | --- | --- | --- | --- | --- | --- | --- | --- |
| Outcomes ^a^ | **N total**  **/Cases** | **None** | **≤1 serving/d** | **1 to 2 serving/d** | **>2serving/d** | **None** | **≤1 serving/d** | **1 to 2 serving/d** | **>2serving/d** | **None** | **≤1 serving/d** | **1 to 2 serving/d** | **>2serving/d** |
| **Excluding self-reported diseases** | | | | | | | | | | | | | |
| Body weight change (kg/year) | **53,692** | ref | -0.04 (0.02) | -0.05 (0.02) | 0.04 (0.03) | ref | 0.02 (0.01) | 0.09 (0.02) | 0.14 (0.04) | ref | -0.08 (0.02) | -0.08 (0.03) | -0.06 (0.05) |
| Waist circumference change (cm/year) | **53,692** | ref | 0.00 (0.02) | 0.01 (0.03) | 0.15 (0.05) | ref | 0.08 (0.02) | 0.20 (0.03) | 0.40 (0.05) | ref | -0.06 (0.02) | -0.05 (0.03) | -0.03 (0.07) |
| Overweight/obesity incidence | **27,756/3,674** | ref | 0.94 (0.88-1.01) | 0.90 (0.81-1.00) | 1.14 (0.99-1.30) | ref | 1.07 (1.01-1.13) | 1.19 (1.08-1.30) | 1.20 (1.04-1.38) | ref | 0.88 (0.82-0.94) | 0.92 (0.83-1.03) | 0.97 (0.78-1.18) |
| Abdominal obesity incidence | **24,947/5,151** | ref | 1.00 (0.94-1.05) | 0.99 (0.91-1.08) | 1.11 (0.99-1.25) | ref | 1.05 (1.00-1.11) | 1.13 (1.04-1.22) | 1.13 (0.99-1.28) | ref | 0.99 (0.94-1.05) | 1.05 (0.96-1.15) | 1.10 (0.93-1.29) |
| **Adjusted for desire to lose weight** | | | | | | | | | | | | | |
| Body weight change (kg/year) | **78,152** | ref | -0.04 (0.01) | -0.03 (0.02) | 0.04 (0.03) | ref | 0.03 (0.01) | 0.09 (0.02) | 0.20 (0.03) | ref | -0.07 (0.02) | -0.07 (0.02) | -0.09 (0.05) |
| Waist circumference change (cm/year) | **78,152** | ref | -0.03 (0.02) | 0.01 (0.03) | 0.13 (0.04) | ref | 0.07 (0.02) | 0.18 (0.03) | 0.39 (0.04) | ref | -0.07 (0.02) | -0.04 (0.03) | -0.02 (0.06) |
| Overweight/obesity incidence | **35,146/4,871** | ref | 0.94 (0.89-0.99) | 0.92 (0.84-1.00) | 1.16 (1.02-1.31) | ref | 1.06 (1.01-1.12) | 1.18 (1.09-1.28) | 1.26 (1.12-1.243) | ref | 0.89 (0.84-0.94) | 0.94 (0.86-1.03) | 1.00 (0.84-1.20) |
| Abdominal obesity incidence | **31,246/6,883** | ref | 1.00 (0.96-1.05) | 1.03 (0.96-1.11) | 1.09 (0.98-1.21) | ref | 1.05 (1.00-1.09) | 1.09 (1.02-1.17) | 1.10 (0.98-1.22) | ref | 0.98 (0.94-1.03) | 1.05 (0.97-1.14) | 1.06 (0.91-1.24) |
| **Excluding desire to lose weight** | | | | | | | | | | | | | |
| Body weight change (kg/year) | **33,741** | ref | -0.03 (0.02) | 0.00 (0.03) | 0.06 (0.04) | ref | 0.02 (0.01) | 0.10 (0.03) | 0.13 (0.04) | ref | -0.06 (0.02) | -0.04 (0.03) | 0.06 (0.05) |
| Waist circumference change (cm/year) | **33,741** | ref | 0.03 (0.02) | 0.12 (0.04) | 0.14 (0.05) | ref | 0.08 (0.02) | 0.18 (0.04) | 0.34 (0.06) | ref | -0.07 (0.02) | -0.05 (0.04) | 0.03 (0.07) |
| Overweight/obesity incidence | **24,815/2,495** | ref | 0.93 (0.86-1.01) | 0.84 (0.74-0.95) | 1.13 (0.96-1.34) | ref | 1.11 (1.03-1.19) | 1.17 (1.04-1.32) | 1.40 (1.15-1.69) | ref | 0.90 (0.83-0.97) | 0.92 (0.81-1.05) | 1.12 (0.90-1.41) |
| Abdominal obesity incidence | **21,854/3,703** | ref | 0.97 (0.91-1.04) | 1.01 (0.91-1.12) | 1.10 (0.95-1.27) | ref | 1.07 (1.01-1.14) | 1.17 (1.05-1.30) | 1.07 (0.89-1.30) | ref | 0.97 (0.91-1.04) | 1.06 (0.95-1.18) | 1.14 (0.94-1.38) |
| ^a^ Results given are mean (SE) or IPR (95%CI).  ^b^ 134 participants did not answer the questions regarding their desire to lose weight (i.e., wish to lose weight).  Model adjusted for age, sex, baseline weight and height (or baseline BMI for overweight/obesity incidence models) or baseline waist circumference (for models with waist circumference or abdominal obesity as outcome), education (categorical), physical activity (continuous), sedentary behavior (continuous), smoking (categorical), alcohol intake (categorical), intakes of fruits, vegetables, legumes, nuts, meat, dairy, sugary foods, potatoes, fats, grains, coffee and tea (g/d) + SSB and either Fruit juice or LNCB (if model LNCB or Fruit juice) history of diseases (diabetes, CVD, hypertension and hypercholesterolemia) and total energy intake (kcal/d).  BMI: Body mass index; SSB: Sugar-sweetened beverages; LNCB: Low/non-calorie beverages; CVD: Cardiovascular diseases; IPR: Incidence proportion ratio. | | | | | | | | | | | | | |

Supplementary Table 4. Adjusted linear associations between SSB and LNCB consumption with weight related outcomes stratified by BMI, sex, and age in the Lifelines Cohort Study

| **Outcomes ^a^** | Total N  Cases N | **SSB (1 serving/day)** | **p-int**^b^ | **LNCB ( 1 serving/day)** | **p-int**^b^ | **Fruit Juice (1 serving/d)** | **p-int**^b^ |
| --- | --- | --- | --- | --- | --- | --- | --- |
| **Body weight change (kg/year)** |  |  |  |  |  |  |  |
| Normal (BMI < 25 kg/m^2^) | 35,202 | 0.04 (0.01) | <.001 | 0.06 (0.01) | .001 | -0.01 (0.01) | .01 |
| Overweight/obese (BMI ≥ 25 kg/m^2^) | 43,084 | 0.00 (0.01) |  | 0.06 (0.01) |  | -0.03 (0.01) |  |
| Women | 46,663 | 0.02 (0.01) | .11 | 0.06 (0.01) | .27 | -0.03 (0.01) | .05 |
| Men | 31,623 | 0.02 (0.01) |  | 0.07 (0.01) |  | -0.01 (0.01) |  |
| Age < 46y | 38,077 | 0.02 (0.01) | .46 | 0.06 (0.01) | .05 | -0.01 (0.01) | .22 |
| Age ≥ 46y | 40,209 | 0.01 (0.01) |  | 0.07 (0.01) |  | -0.04 (0.01) |  |
| **Waist circumference change (cm/year)** |  |  |  |  |  |  |  |
| Normal weight | 35,202 | 0.04 (0.01) | .13 | 0.08 (0.01) | .06 | 0.01 (0.02) | .11 |
| Overweight/obese | 43,084 | 0.03 (0.01) |  | 0.10 (0.01) |  | -0.01 (0.02) |  |
| Women | 46,663 | 0.04 (0.02) | .32 | 0.13 (0.01) | .87 | -0.01 (0.02) | .13 |
| Men | 31,623 | 0.02 (0.01) |  | 0.09 (0.01) |  | 0.01 (0.01) |  |
| Age < 46y | 38,077 | 0.04 (0.01) | .61 | 0.11 (0.01) | .71 | 0.00 (0.02) | .52 |
| Age ≥ 46y | 40,209 | 0.04 (0.02) |  | 0.11 (0.01) |  | 0.00 (0.02) |  |
| **Overweight/obesity incidence** |  |  |  |  |  |  |  |
| Women | 23,617/3,131 | 1.02 (0.97-1.06) | .76 | 1.07 (1.04-1.11) | .30 | 0.98 (0.92-1.03) | .30 |
| Men | 11,585/1,753 | 1.04 (1.00-1.09) |  | 1.11 (1.05-1.16) |  | 1.04 (0.97-1.10) |  |
| Age < 46y | 19,952/2,720 | 1.02 (0.99, 1.06) | .52 | 1.08 (1.05, 1.12) | .34 | 1.00 (0.95-1.08) | .97 |
| Age ≥ 46y | 15,250/2,164 | 1.06 (0.99, 1.13) |  | 1.09 (1.03-1.14) |  | 1.01 (0.93-1.08) |  |
| **Abdominal obesity incidence** |  |  |  |  |  |  |  |
| Women | 15,689/4,237 | 1.02 (0.98-1.06) | .71 | 1.03 (1.00-1.06) | .04 | 1.03 (0.99-1.08) | .99 |
| Men | 15,603/2,659 | 1.01 (0.97-1.05) |  | 1.06 (1.03-1.10) |  | 1.02 (0.98-1.08) |  |
| Age < 46y | 18,887/3,697 | 1.02 (0.99, 1.05) | .33 | 1.04 (1.01-1.07) | .75 | 1.04 (1.00-1.08) | .88 |
| Age ≥ 46y | 12,405/3199 | 1.03 (0.98, 1.08) |  | 1.05 (1.01-1.09) |  | 1.01 (0.96-1.07) |  |
| ^a^ Results given are mean (SE) or IPR (95%CI).^b^ P-values for the interaction term of beverage consumption and BMI, sex or age categories in corresponding models.  All models were adjusted by age, sex, baseline weight and height (or baseline BMI for overweight/obesity) or baseline waist circumference (for models with waist circumference and abdominal obesity models as outcome), education (categorical), physical activity (continuous), sedentary behavior (continuous), smoking (categorical), alcohol intake (categorical), intakes of fruits, vegetables, legumes, nuts, meat, dairy, sugary foods, potatoes, fats, grains, coffee and tea (g/d), LNCB, SSB, history of diseases (diabetes, CVD, hypertension, and hypercholesterolemia) and total energy intake (kcal/d).  BMI: Body mass index; SSB: Sugar-sweetened beverages; LNCB: Low/No-calorie beverage; CVD: Cardiovascular diseases; IPR: Incidence proportion ratio. | | | | | | | |

Supplementary Table 5. Adjusted associations between LNCB and fruit juice intake categories and weight-related outcomes stratified by BMI, sex and age in the Lifelines Cohort Study

|  |  | **SSB** | | | |  | **LNCB** | | | | | **Fruit Juice** | | | | |
| --- | --- | --- | --- | --- | --- | --- | --- | --- | --- | --- | --- | --- | --- | --- | --- | --- |
| **Outcomes** ^a^ | N total/Cases | None | ≤1 serving/d | 1 to 2 servings/d | >2servings/d | p-int^b^ | None | ≤1 serving/d | 1 to 2 serving/d | >2servings/d | p-int^b^ | None | ≤1 serving/d | 1 to 2 servings/d | >2servings/d | p-int^b^ |
| **Body weight change** | | | | | | | | | | | | | | | | |
| Normal weight | 35,202 | ref | -0.05 (0.02) | -0.03 (0.02) | 0.07 (0.03) | <.001 | ref | 0.02 (0.01) | 0.11 (0.02) | 0.15 (0.04) | <.001 | ref | -0.10 (0.02) | -0.06 (0.03) | -0.03 (0.05) | <.01 |
| Overweight/obese | 43,084 | ref | -0.03 (0.02) | -0.04 (0.03) | 0.02 (0.05) |  | ref | 0.03 (0.02) | 0.07 (0.03) | 0.23 (0.04) |  | ref | -0.06 (0.02) | -0.08 (0.04) | -0.15 (0.07) |  |
| Women | 46,663 | ref | -0.06 (0.02) | -0.06 (0.03) | 0.00 (0.05) | <.001 | ref | 0.03 (0.02) | 0.09 (0.03) | 0.20 (0.04) | .54 | ref | -0.10 (0.02) | -0.10 (0.03) | -0.13 (0.07) | .03 |
| Men | 31,623 | ref | 0.01 (0.02) | 0.01 (0.03) | 0.09 (0.04) |  | ref | 0.03 (0.02) | 0.10 (0.03) | 0.20 (0.04) |  | ref | -0.02 (0.02) | -0.02 (0.03) | -0.04 (0.06) |  |
| Age < 46y | 38,077 | ref | -0.08 (0.02) | -0.06 (0.03) | 0.02 (0.04) | .58 | ref | 0.01 (0.02) | 0.09 (0.03) | 0.17 (0.04) | .16 | ref | -0.09 (0.02) | -0.07 (0.03) | -0.11 (0.06) | .12 |
| Age >= 46y | 40,209 | ref | -0.01 (0.02) | -0.01 (0.03) | 0.02 (0.06) |  | ref | 0.04 (0.02) | 0.09 (0.03) | 0.23 (0.04) |  | ref | -0.06 (0.02) | -0.08 (0.03) | -0.09 (0.08) |  |
| **Waist circumference change** | | | | | | | | | | | | | | | | |
| Normal weight | 35,202 | ref | 0.02 (0.02) | 0.07 (0.04) | 0.10 (0.05) | <.001 | ref | 0.09 (0.02) | 0.17 (0.04) | 0.26 (0.06) | .09 | ref | -0.03 (0.02) | -0.00 (0.04) | 0.01 (0.08) | .21 |
| Overweight/obese | 43,084 | ref | -0.04 (0.02) | -0.04 (0.04) | 0.10 (0.06) |  | ref | 0.04 (0.02) | 0.14 (0.03) | 0.35 (0.05) |  | ref | -0.07 (0.02) | -0.06 (0.04) | -0.06 (0.08) |  |
| Women | 46,663 | ref | -0.04 (0.02) | -0.04 (0.04) | 0.13 (0.06) | <.01 | ref | 0.11 (0.02) | 0.22 (0.04) | 0.47 (0.05) | .76 | ref | -0.07 (0.02) | -0.06 (0.04) | -0.05 (0.09) | .20 |
| Men | 31,623 | ref | 0.00 (0.02) | 0.04 (0.03) | 0.07 (0.05) |  | ref | 0.05 (0.02) | 0.16 (0.03) | 0.28 (0.05) |  | ref | -0.02 (0.02) | 0.01 (0.04) | 0.00 (0.07) |  |
| Age < 46y | 38,077 | ref | -0.01 (0.03) | 0.01 (0.04) | 0.13 (0.051 | .59 | ref | 0.08 (0.02) | 0.18 (0.04) | 0.42 (0.05) | .25 | ref | -0.05 (0.03) | -0.04 (0.04) | -0.05 (0.07) | .44 |
| Age ≥ 46y | 40,209 | ref | -0.04 (0.02) | 0.02 (0.04) | 0.09 (0.08) |  | ref | 0.08 (0.02) | 0.21 (0.04) | 0.35 (0.06) |  | ref | -0.07 (0.02) | -0.03 (0.04) | 0.06 (0.10) |  |
| **Overweight/obesity incidence** | | | | | | | | | | | | | | | | |
| Women | 23,617/3,131 | ref | 0.90 (0.85-0.97) | 0.89 (0.79-1.00) | 1.09 (0.92-1.29) | .79 | ref | 1.08 (1.01-1.15) | 1.19 (1.08-1.31) | 1.24 (1.06-1.45) | .62 | ref | 0.86 (0.80-0.92) | 0.89 (0.78-1.00) | 0.94 (0.73-1.20) | .76 |
| Men | 11,585/1,753 | ref | 1.02 (0.92-1.14) | 0.97 (0.84-1.12) | 1.29 (1.07-1.55) |  | ref | 1.03 (0.94-1.12) | 1.16 (1.00-1.34) | 1.28 (1.04-1.57) |  | ref | 0.97 (0.88-1.07) | 1.04 (0.90-1.20) | 1.09 (0.83-1.42) |  |
| Age < 46y | 19,952/2,720 | ref | 0.85 (0.79-0.92) | 0.85 (0.76-0.95) | 1.06 (0.92-1.23) | .38 | ref | 1.03 (0.95-1.10) | 1.17 (1.05-1.29) | 1.23 (1.06-1.43) | .26 | ref | 0.83 (0.77-0.90) | 0.87 (0.78-0.98) | 0.93 (0.75-1.15) | .81 |
| Age ≥ 46y | 15,250/2,164 | ref | 1.03 (0.95-1.11) | 0.99 (0.84-1.17) | 1.27 (0.94-1.71) |  | ref | 1.10 (1.02-1.18) | 1.19 (1.04-1.36) | 1.27 (1.01-1.59) |  | ref | 0.95 (0.88-1.03) | 1.03 (0.88-1.20) | 1.14 (0.81-1.61) |  |
| **Abdominal obesity incidence** | | | | | | | | | | | | | | | | |
| Women | 15,689/4237 | ref | 0.98 (0.92-1.03) | 0.96 (0.87-1.06) | 1.05 (0.91-1.22) | .90 | ref | 1.08 (1.02-1.13) | 1.14 (1.04-1.24) | 1.07 (0.92-1.24) | .22 | ref | 1.00 (0.94-1.06) | 1.03 (0.93-1.14) | 1.10 (0.91-1.34) | .92 |
| Men | 15,603/2,659 | ref | 1.04 (0.96-1.13) | 1.07 (0.96-1.21) | 1.07 (0.92-1.26) |  | ref | 1.05 (0.98-1.12) | 1.10 (0.98-1.23) | 1.19 (1.00-1.41) |  | ref | 1.00 (0.92-1.09) | 1.10 (0.98-1.25) | 1.01 (0.77-1.28) |  |
| Age < 46y | 18,887/3,697 | ref | 1.02 (0.95-1.09) | 1.01 (0.92-1.12) | 1.09 (0.96-1.24) | .57 | Ref | 1.06 (0.99-1.12) | 1.10 (1.00-1.20) | 1.13 (0.98-1.30) | .78 | ref | 0.98 (0.91-1.05) | 1.02 (0.91-1.13) | 1.00 (0.83-1.21) | .41 |
| Age ≥ 46y | 12,405/3,199 | ref | 0.98 (0.92-1.04) | 1.05 (0.93-1.18) | 1.03 (0.82-1.30) |  | ref | 1.07 (1.01-1.14) | 1.14 (1.02-1.27) | 1.13 (0.93-1.36) |  | ref | 1.00 (0.93-1.06) | 1.09 (0.96-1.23) | 1.17 (0.91-1.50) |  |
| ^a^ Results given are mean (SE) or IPR (95%CI).^b^ P-value for the interaction term of beverage consumption and BMI, sex, or age categories.  All models were adjusted by age, sex, baseline weight and height (or baseline BMI for overweight/obesity) or baseline waist circumference (for models with waist circumference and abdominal obesity models as outcome), education (categorical), physical activity (continuous), sedentary behavior (continuous), smoking (categorical), alcohol intake (categorical), intakes of fruits, vegetables, legumes, nuts, meat, dairy, sugary foods, potatoes, fats, grains, coffee and tea (g/d), LNCB, SSB, history of diseases (diabetes, CVD, hypertension, and hypercholesterolemia) and total energy intake (kcal/d) (model 3).  BMI: Body mass index; SSB: Sugar-sweetened beverages; LNCB: Low/No-calorie beverage; CVD: Cardiovascular diseases; IPR: Incidence proportion ratio. | | | | | | | | | | | | | | | | |

Supplementary Table 6. Adjusted substitution analyzes of beverages replacements with weight-related outcomes stratified by BMI, sex, and age ^a^

| **Outcome** | **N total/Cases** | **Substitution of SSB with LNCB** | **Substitution of SSB with Fruit Juice** | **Substitution of SSB with water** ^d^ | **Substitution of LNCB with water** |
| --- | --- | --- | --- | --- | --- |
| **Weight change (kg/y)** ^b^ |  |  |  |  |  |
| Normal weight | 35,202 | 0.02 (0.01) | -0.04 (0.01) | -0.02 (0.02) | -0.05 (0.02) |
| Overweight/obese | 43,084 | 0.06 (0.01) | -0.03 (0.02) | -0.02 (0.02) | -0.05 (0.02) |
| Women | 46,663 | 0.04 (0.01) | -0.05 (0.02) | -0.02 (0.02) | -0.04 (0.02) |
| Men | 31,623 | 0.05 (0.01) | -0.02 (0.02) | -0.02 (0.02) | -0.06 (0.02) |
| Age < 46y | 38,077 | 0.04 (0.01) | -0.03 (0.02) | -0.02 (0.02) | -0.03 (0.02) |
| Age >= 46y | 40,209 | 0.06 (0.02) | -0.05 (0.02) | -0.02 (0.02) | -0.08 (0.02) |
| **Waist circumference change (cm/y)** ^b^ |  |  |  |  |  |
| Normal weight | 35,202 | 0.05 (0.02) | -0.03 (0.02) | -0.01 (0.03) | -0.04 (0.03) |
| Overweight/obese | 43,084 | 0.07 (0.02) | -0.04 (0.02) | -0.07 (0.03) | -0.08 (0.02) |
| Women | 46,663 | 0.09 (0.02) | -0.05 (0.03) | -0.06 (0.03) | -0.09 (0.02) |
| Men | 31,623 | 0.07 (0.02) | -0.02 (0.02) | -0.02 (0.02) | -0.06 (0.02) |
| Age < 46y | 38,077 | 0.08 (0.02) | -0.04 (0.02) | -0.04 (0.02) | -0.06 (0.02) |
| Age >= 46y | 40,209 | 0.08 (0.02) | -0.04 (0.03) | -0.05 (0.03) | -0.09 (0.02) |
| **Overweight incidence** ^c^ |  |  |  |  |  |
| Women | 23,617/3,131 | 1.05 (1.00-1.11) | 0.96 (0.89-1.03) | 1.02 (0.93-1.11) | 0.93 (0.87-0.99) |
| Men | 11,585/1,753 | 1.08 (1.01-1.14) | 0.99 (0.92-1.07) | 0.99 (0.89-1.10) | 0.86 (0.76-0.97) |
| Age < 46y | 19,952/2,720 | 1.06 (1.01-1.11) | 0.98 (0.91-1.04) | 1.04 (0.95-1.12) | 0.94 (0.87-1.01) |
| Age >= 46y | 15,250/2,164 | 1.05 (0.96-1.13) | 0.96 (0.85-1.06) | 0.92 (0.79-1.05) | 0.86 (0.76-0.96) |
| **Abdominal obesity**  **incidence** ^c^ |  |  |  |  |  |
| Women | 15,689/4,237 | 1.02 (0.97-1.06) | 1.02 (0.96-1.0) | 1.02 (0.95-1.09) | 0.97 (0.91-1.03) |
| Men | 15,603/2,659 | 1.05 (1.00-1.10) | 1.01 (0.95-1.08) | 1.03 (0.95-1.10) | 0.97 (0.90-1.04) |
| Age < 46y | 18,887/3,697 | 1.02 (0.98-1.06) | 1.02 (0.96-1.07) | 1.05 (0.99-1.11) | 0.97 (0.91-1.03) |
| Age >= 46y | 12,405/3,199 | 1.02 (0.96-1.08) | 0.99 (0.91-1.06) | 0.97 (0.88-1.07) | 0.97 (0.91-1.04) |
| ^a^ Fully-adjusted models without total energy (model 3) with total beverages + individual beverage used for replacement (i.e. y= intercept + B1(total beverages) + B2(LNCB) + B3(Fruit Juice) + B(covariates) + ε)  ^b^ Results given are mean (SE) from multiple regression models.  ^c^ Results given as IPR (95%CI).  ^d^ Substitution analyzes with water could only be performed on a sub-sample size of N=22,859 participants (n = 10,285 participants with normal-BMI and n = 12,574 participants with overweight/obesity)  All p-interactions were > .05, except in the substitution of SSB by LNCB for BMI categories (p<.001) with weight as outcome and sex (p = .04) with abdominal obesity as outcome; and in the substitution of SSB by fruit juice with BMI (P=.01) and sex (p = .05) both with weight as outcome in the substitution analysis of SSB with fruit juice.  BMI: Body mass index; SSB: Sugar-sweetened beverages; LNCB: Low/No-calorie beverage; IPR: Incidence proportion ratio. | | | | | |
